# Bivalent Oral Vaccine Using Attenuated *Salmonella* Gallinarum Delivering HA and NA-M2e Confers Dual Protection Against H9N2 Avian Influenza and Fowl Typhoid in Chickens

**DOI:** 10.3390/vaccines13080790

**Published:** 2025-07-25

**Authors:** Muhammad Bakhsh, Amal Senevirathne, Jamal Riaz, Jun Kwon, Ram Prasad Aganja, Jaime C. Cabarles, Sang-Ik Oh, John Hwa Lee

**Affiliations:** 1 College of Veterinary Medicine, Jeonbuk National University, Specialized Campus, Iksan 54596, Republic of Korea; mbakhsh90@jbnu.ac.kr (M.B.); amal.senevirathne@jbnu.ac.kr (A.S.); 202559026@jbnu.ac.kr (J.R.); kjun1002@jbnu.ac.kr (J.K.); ramaganja@jbnu.ac.kr (R.P.A.); 2 Central Department of Biotechnology, Tribhuvan University, Kirtipur 44618, Nepal; 3 College of Agriculture, Resources and Environmental Science, Central Philippine University, Jaro, Iloilo City 5000, Philippines; jccabarlesjr@cpu.edu.ph

**Keywords:** H9N2, fowl typhoid, *Salmonella*, dual expression system, immune response, bivalent vaccine

## Abstract

**Background:** Fowl typhoid (FT), a septicemic infection caused by *Salmonella* Gallinarum (SG), and H9N2 avian influenza are two economically important diseases that significantly affect the global poultry industry. **Methods:** We exploited the live attenuated *Salmonella* Gallinarum (SG) mutant JOL3062 (SG: ∆*lon* ∆*pagL* ∆*asd*) as a delivery system for H9N2 antigens to induce an immunoprotective response against both H9N2 and FT. To enhance immune protection against H9N2, a prokaryotic and eukaryotic dual expression plasmid, pJHL270, was employed. The hemagglutinin (HA) consensus sequence from South Korean avian influenza A virus (AIV) was cloned under the Ptrc promoter for prokaryotic expression, and the B cell epitope of neuraminidase (NA) linked with matrix protein 2 (M2e) was placed for eukaryotic expression. In vitro and in vivo expressions of the H9N2 antigens were validated by qRT-PCR and Western blot, respectively. **Results:** Oral immunization with JOL3121 induced a significant increase in SG and H9N2-specific serum IgY and cloacal swab IgA antibodies, confirming humoral and mucosal immune responses. Furthermore, FACS analysis showed increased CD4+ and CD8+ T cell populations. On day 28 post-immunization, there was a substantial rise in the hemagglutination inhibition titer in the immunized birds, demonstrating neutralization capabilities of immunization. Both IFN-γ and IL-4 demonstrated a significant increase, indicating a balance of Th1 and Th2 responses. Intranasal challenge with the H9N2 Y280 strain resulted in minimal to no clinical signs with significantly lower lung viral titer in the JOL3121 group. Upon SG wildtype challenge, the immunized birds in the JOL3121 group yielded 20% mortality, while 80% mortality was recorded in the PBS control group. Additionally, bacterial load in the spleen and liver was significantly lower in the immunized birds. **Conclusions:** The current vaccine model, designed with a host-specific pathogen, SG, delivers a robust immune boost that could enhance dual protection against FT and H9N2 infection, both being significant diseases in poultry, as well as ensure public health.

## 1. Introduction

The global poultry industry faces persistent challenges from infectious diseases that significantly impact animal health, productivity, and overall food security. Among these, lowpathogenic avian influenza virus (LPAIV) subtype H9N2 and fowl typhoid (FT) caused by *Salmonella* Gallinarum (SG) are two major threats due to their widespread occurrence, economic consequences, and zoonotic potential [1,2,3]. Over the past two decades, H9N2 has become enzootic in poultry across Asia, the Middle East, and Africa, leading to severe financial losses by compromising production efficiency and increasing mortality through enhanced susceptibility to co-infections [4,5,6,7]. Chickens, turkeys, ducks, and quails represent primary hosts, exhibiting mild to severe respiratory signs depending on host immunity and environmental factors. Although H9N2 infections in humans are rare and generally asymptomatic, they remain a cause for global concern as the virus continues to undergo genetic evolution and reassortment, increasing the risk of a future pandemic [8,9,10]. Conventional vaccination strategies for H9N2 primarily rely on inactivated whole-virus vaccines formulated with adjuvants. While these vaccines are effective in reducing clinical signs and viral shedding in the short term, their efficacy is frequently compromised by the virus’s rapid antigenic drift [11,12,13,14]. Consequently, poultry flocks often require repeated immunizations aligned with emerging strain profiles, adding to labor, cost, and logistical burdens. Moreover, these injectable vaccines are typically administered individually, further complicating large-scale immunization programs, especially in resource-limited settings.

Fowl typhoid, on the other hand, continues to be a major concern in poultry farming systems, particularly in regions with inadequate biosecurity. It results in systemic infection, high mortality, and considerable economic losses [15,16,17,18]. Current control measures include the use of antibiotics, strict biosecurity regulations, and vaccination. Vaccination is one of the most effective strategies for controlling FT, just as it is for managing other infectious diseases [19,20]. Several vaccine strategies against FT have been developed, including live-attenuated, killed, and subunit vaccines [21]. The commercial live attenuated semi-rough SG9R vaccine has been used for over six decades to control FT. However, it has been reported to cause mild systemic infection and bacterial persistence for several weeks in young birds [19,22]. Furthermore, it has been associated with limited protection, residual virulence, and vertical transmission [23,24,25]. Of particular concern is the immunosuppressive effect induced by *Salmonella* infection, which can exacerbate the severity of co-infections, including those caused by avian influenza viruses. Field observations have demonstrated that co-infection with SG and H9N2 leads to aggravated disease manifestations, underscoring the urgent need for integrated vaccination strategies.

To address these challenges, novel immunization approaches are being explored, one of which involves the use of attenuated SG as a live bacterial vector for delivering recombinant antigens. This strategy leverages the bacterium’s host specificity and its natural ability to colonize and disseminate through the gastrointestinal-associated lymphoid tissue (GALT) of poultry. Once administered orally, the attenuated SG vector traffics through Peyer’s patches and other mucosal immune sites, efficiently stimulating both mucosal and systemic immunity. Unlike inactivated vaccines that primarily induce systemic IgY responses, live bacterial vectors are capable of eliciting robust mucosal IgA responses, which are critical for combating respiratory pathogens such as H9N2 [26,27,28,29]. One of the key advantages of using SG as a vector is its suitability for oral administration, enabling mass vaccination through drinking water or feed, an approach that significantly reduces labor and handling stress. This is particularly important in large-scale poultry operations where logistical efficiency is paramount. The potential for incorporating multiple antigens within a single vector also makes this platform ideal for developing combination vaccines.

In the context of avian influenza, hemagglutinin (HA) remains the primary target for vaccine-induced neutralizing antibodies. It is a surface glycoprotein responsible for viral attachment to host cell sialic acid receptors, making it essential for viral entry. Structurally stable, recombinant trimeric HA proteins have demonstrated the ability to induce cross-protective immunity across various influenza strains [30,31,32]. Neuraminidase (NA), another surface antigen, plays a supportive role in viral release and spread. NA-inhibiting antibodies contribute significantly to overall protection and help reduce viral load [33]. Additionally, internal conserved antigens such as the extracellular domain of matrix protein 2 (M2e) are increasingly being used to broaden vaccine coverage against antigenically diverse strains [34,35,36]. These antigens are relatively conserved and capable of eliciting cross-reactive immune responses, offering a promising solution to the limitations of strain-specific vaccines.

In our study, we developed a dual-faceted SG-mediated vaccine platform capable of delivering protective immunity against both H9N2 and FT. We engineered an attenuated SG strain (Δ*lon* Δ*pagL* Δ*asd*) to carry a dual expression plasmid, pJHL270, designed for simultaneous prokaryotic and eukaryotic expression of recombinant influenza antigens. The antigenic construct consisted of a consensus HA sequence derived from eight representative H9N2 strains of the Y280 lineage and a B cell epitope of NA linked with the conserved M2e sequence. This design was intended to maximize immunogenic breadth and durability against circulating and emerging H9N2 variants. Following oral immunization, we evaluated the induced immune responses, focusing on both SG-specific and H9N2-specific antibody profiles. The vaccine elicited strong mucosal and systemic responses, as well as elevated Th1 and Th2 cytokine expression. Furthermore, challenge trials with virulent SG and the H9N2 Y280 strain confirmed the protective efficacy of the vaccine, as evidenced by reduced mortality, diminished clinical symptoms, and significantly lower pathogen loads in vaccinated birds.

In conclusion, the integration of H9N2 antigens within an SG-based live vector represents a highly promising advancement in poultry vaccine development. This approach not only addresses the limitations of conventional vaccination but also offers practical advantages in terms of administration, cost, and immune effectiveness. By simultaneously protecting against two economically significant and immunologically distinct pathogens, the dual-faceted SG-mediated vaccine platform offers a strategic and sustainable solution for improving poultry health and productivity in endemic regions.

## 2. Materials and Methods

### 2.1. Bacterial Strains, Plasmids, And Primers

The bacterial strains, plasmids, and primers used in this study are listed in Table 1. All bacterial strains were cultured in Luria–Bertani (LB) medium (BD, Franklin Lakes, NJ, USA) at 37 °C, with the appropriate selection markers as needed. The attenuated SG strain, which has deletions of the *lon*, *pagL*, and *asd* genes (JOL3062), was developed as a delivery vector for this study. The JOL3062 strain was engineered from JOL2997 [3] by deleting the *asd* gene, using the λ-red recombination technique as described elsewhere [1], with some modifications. All strains are stored at –80 °C in the laboratory stock of the College of Veterinary Medicine, Jeonbuk National University, South Korea.

### 2.2. Vaccine Design

A bivalent SG-based live attenuated vaccine was designed against H9N2 and FT. The HA gene sequences of the H9N2 Y280 influenza A virus strains from South Korea (2000–2021) were obtained from the NCBI database. Retrieved sequences were aligned and analyzed using multiple alignment tools (EMBL-EBI, ClustalW v2.1) to derive the consensus sequence and optimized for *Salmonella* expression. The current HA antigen structure contains 533 AA, including both HA1 and HA2 portions for improved structural integrity. In addition to this, a second antigen was designed by combining the B cell epitope of neuraminidase (NA) with the conserved extracellular domain of matrix protein 2 (M2e). Briefly, a Kozak sequence was included to enhance translational efficiency in the eukaryotic expression system, followed by a signal peptide sequence to facilitate secretion of the expressed antigen. The antigenic portion consisting of the B cell epitope of NA (121–340 amino acids) was fused via a furin cleavage to the M2e. This innovative strategy aimed to enhance immunogenicity by presenting multiple conserved influenza antigens in a single vaccine construct to achieve effective protection against H9N2.

The dual expression plasmid vector pJHL270 was previously developed by our lab from the pMMp65 vector. A synthetic gene fragment was designed for a eukaryotic expression system, incorporating a CMV enhancer, CMV promoter, multiple cloning site (MCS), and a poly A sequence (Cosmogenetech, Seoul, Republic of Korea). Both the synthesized fragment and the pMMp65 vector were double-digested using XbaI and BspHI restriction enzymes. The resulting digested products were ligated to construct the recombinant plasmid, which was designated as pJHL270. The consensus HA sequence was expressed by the Ptrc constitutive promoter of pJHL270, designed for prokaryotic expression after codon optimization for efficient *Salmonella* expression. The NA-M2e construct was kept under the control of the CMV promoter and optimized for eukaryotic expression. The resultant plasmid (pJHL270 containing pHA and eNA-M2e) was electroporated at 2.5 kV (BTX, Harvard Apparatus, Holliston, MA, USA) into the SG strain, JOL3062 (SG: Δ*lon* Δ*pagL* Δ*asd*), yielding the vaccine strain, JOL3121. The empty vector pJHL270 was also electroporated to create the vector control strain, JOL3122.

Individual H9N2 antigens were cloned into the pET28a (+) expression vector, and the resulting recombinant plasmids were transformed into BL21 (DE3) competent cells. Protein expression was induced with 1 mM IPTG at 37 °C for 4 h. The expressed antigens were analyzed by SDS-PAGE and purified under denaturing conditions using nickel-NTA affinity chromatography (Takara, Shiga, Japan). The purified proteins were then dialyzed against phosphate-buffered saline (PBS) and subsequently used to produce polyclonal antibodies in New Zealand white rabbits, aged two months.

### 2.3. Confirmation of Clones And Antigen mRNA Transcription In Vitro

Clonal verification and the detection of open reading frames (ORFs) were performed using PCR. The antigen expression by the vaccine strain was validated by qRT-PCR. To confirm the prokaryotic expression, JOL3121 was grown to mid-log phase. Cells were collected by centrifugation, and the total RNA was extracted from the cells using the Trizol method (GeneAll, Hanam-si, Gyeonggi-do, Republic of Korea) following the manufacturer’s instructions. cDNA was synthesized by mixing 1 μg of RNA with reverse transcriptase 5 × premix (Elpis Biotech, Daejeon, Republic of Korea) and oligo (dT) and used in qRT-PCR-based expression analysis. For eukaryotic expression of the antigen, RAW 264.7 cells were cultured in 6-well plates at a density of 1 × 10^6^ cells per well in Dulbecco’s modified Eagle medium (DMEM) (Serana Europe, Pessin, Germany), supplemented with 10% fetal bovine serum (FBS) and 1% broad-spectrum antibiotics. The cells were transfected with JOL3121 and JOL3122 for 4 hours. After infection, the medium was replaced with DMEM containing 100 μg/mL gentamicin, and the cells were further incubated at 37 °C with 5% CO_2_ for 24 h. Total RNA was isolated, cDNA was synthesized, and qRT-PCR was performed as described earlier.

### 2.4. Animals And Ethics

One-day-old, specific-pathogen-free (SPF), female brown nick layer chickens were purchased from the Corporation of Joint Hatchery, Pyeongtaek, South Korea. All bird experimentation was approved by Jeonbuk National University and handled according to the Animal Ethics Committee (NON2023-135-001) guidelines under the Korean Council on Animal Care and the Korean Animal Protection Law enacted in 2007. The chickens were provided food and water *ad libitum*. All the chickens were regularly monitored for behavioral and physiological changes.

### 2.5. Chicken Inoculation And Confirmation of Antigen Expression In Vivo

The expression of HA, NA, and M2e was verified in vivo. Chickens (*n* = 3 per group) were single immunized orally with JOL3121 and JOL3122 (vector control), each containing 1 × 10^8^ CFU/200 µL. At day 3 post-immunization, spleen tissues were harvested, homogenized, and treated with PRO-PREP™ Protein Extraction Solution (iNtRON Biotechnology, Seongnam-si, Republic of Korea), following the manufacturer’s instructions. The protein concentration was measured using the Bradford assay [37]. Approximately 30 µg of proteins from the treatment group, along with the vector control, were loaded, separated by SDS-PAGE, and transferred to a PVDF membrane (Immobilon-P; Millipore, Cork, Ireland). The membrane was treated with 5% skim milk solution for 1 hour at 37 °C to block unsaturated sites. After washing three times with PBS, the membrane was treated with polyclonal antibodies prepared by our lab in New Zealand white rabbits (1:500 dilution) against HA, NA, and M2e and incubated overnight at 4 °C. HRP-conjugated antirabbit IgG antibodies (1:5000 dilution) (Cat. No. 4030-05, SouthernBiotech, Birmingham, AL, USA) were applied for 1 hour at 37 °C. The expected band size was detected by adding Western blotting substrate, WESTSAVE Gold (Abfrontier Co., Ltd., Seoul, Republic of Korea), and recorded for further analysis.

### 2.6. Immunization And Challenge

One-month-old, female nick layer brown chickens were divided into four experimental groups (n = 20 per group), as indicated in Table 2. Each bird was immunized orally with JOL3121 or JOL3122, consisting of 1 × 10^8^ CFU/200 μL of PBS per bird. Additionally, one group received only PBS as a placebo, while the other group remained a naïve control. Two weeks after the primary immunization, booster doses were administered via the same route of administration. Serum and cloacal swab samples were collected from randomly selected birds (*n* = 5) two weeks after the primary and booster vaccinations. Furthermore, two weeks after the booster inoculation, peripheral blood mononuclear cells (PBMCs) were harvested using density gradient centrifugation with Ficoll-Paque PLUS density gradient medium (Cytiva, Uppsala, Sweden) and processed for FACS analysis and qRT-PCR. Two weeks following the booster immunization, half of the birds from each group were moved to new cages and intranasally challenged with 1 × 10^6^ EID_50_/100 μL of H9N2 avian influenza A (Y280 strain). The remaining half of the birds were orally challenged with wild-type SG (JOL422) at a concentration of 1 × 10^6^ CFU/200 μL per bird. Five birds from each H9N2-challenged group were euthanized on days 3 and 7 post-challenge for lung tissue viral titration and comprehensive histological analysis. On day 7 after SG challenge, five birds from each group were euthanized to assess bacterial load in the liver and spleen. Moreover, spleen, liver, and cecum tissues were collected for hematoxylin and eosin staining. In order to derive the Kaplan–Meier survival curve for SG challenge, 30 birds (*n* = 10 per group) were maintained separately and subjected to a complete immunization scheme and challenge. The groups utilized here included PBS, JOL3122, and JOL3121, and the corresponding mortality was recorded daily until day 14 post-challenge.

### 2.7. Enzyme-Linked Immunosorbent Assay (ELISA)

Indirect ELISA was performed to measure SG-specific and H9N2-specific systemic IgY and mucosal IgA in serum and cloacal swab samples collected at days 14 and 28 post-immunization. Briefly, 96-well plates were coated with SG crude soluble protein or 0.1% formalin-inactivated H9N2 virus in 0.5 M Na_2_CO_3_/NaHCO_3_ (pH 9.6) buffer and incubated at 4 °C overnight. At room temperature, the plates were blocked with 5% skim milk as a blocking solution for one hour. Serum (1:100 dilution) and cloacal swab samples (undiluted) were added and incubated at 4 °C overnight. Then the wells were treated with HRP-conjugated goat antichicken antibodies (Bethyl Laboratories, Montgomery, TX, USA) with appropriate dilutions. O-phenylenediamine dihydrochloride (Sigma, St. Louis, MI, USA) was added to initiate the enzymatic reaction and stopped with 2N sulfuric acid (50 µL/well). The absorbance at 492 nm was measured using an ELISA spectrophotometer (Tecan).

### 2.8. Hemagglutination Inhibition (HI) Assay

Two weeks after the booster immunization, serum samples were collected from the immunized birds and underwent heat inactivation at 56 °C for 30 min. The hemagglutination inhibition (HI) assay was performed to assess the antibody response to H9N2 infection as described by the World Organization for Animal Health in 2008 [38]. Briefly, a microplate with V-bottomed wells was used for the HI assay. The serum samples were two-fold serially diluted with 25 μL of PBS and incubated with an equal volume of H9N2 Y280 virus containing four hemagglutinating units (HAUs) at room temperature for 30 minutes. Then, 50 μL of 0.75% chicken red blood cells (RBCs) was introduced to each well and incubated again for 30 minutes at room temperature. The HI titer was measured based on log2 of the reciprocal of the highest serum dilution inhibiting the RBCs’ hemagglutination.

### 2.9. Fluorescence-Activated Cell Sorting (FACS) Analysis

The differentiation of T lymphocyte subpopulations induced by immunization was assessed in peripheral blood mononuclear cells (PBMCs) collected 2 weeks after the booster immunization. The PBMCs were isolated from the blood using Ficoll-Paque PLUS density gradient medium (Cytiva, Uppsala, Sweden) following the manufacturer’s instructions. The cells were seeded at 1 × 10^6^ cells per well in 96-well plates and stimulated either with wild-type SG crude protein or 0.1% formalin-inactivated H9N2 Y280 virus. After 2 days of incubation at 37 °C with 5% CO_2_, the cells were stained with fluorescently labeled anti-CD3-FITC (Cat: 8200-02, Southern Biotech, Birmingham, AL, USA), anti-CD4-AF700 (Cat: 8210-27, Southern Biotech, Birmingham, AL, USA), and anti-CD8a-PE (Cat: 8220-09, Southern Biotech, Birmingham, AL, USA) for 30 minutes at 4 °C. The data were acquired from the stained cells using a MacsQuant flow cytometer (Miltenyi Biotec, Bergisch Gladbach, Germany).

### 2.10. Assessment of Cytokine Expression by Quantitative Real-Time PCR (qRT-PCR)

The PBMCs were isolated and seeded in 12-well plates at 1 × 10^6^ cells per well and stimulated either with wild-type SG crude protein or formalin-inactivated H9N2 Y280 virus. After 2 days of incubation at 37 °C with 5% CO_2_, total RNA was extracted following the manufacturer’s instructions (GeneAll, Seoul, Republic of Korea), and cDNA was synthesized (Elpis Biotech, Daejeon, Republic of Korea). The mRNA expression levels of IFN-γ and IL-4 cytokines were quantified by qRT-PCR using SYBR Green Master Mix (Elpis Biotech, Republic of Korea), and primers are listed in Table 1. The changes in mRNA levels were determined by the 2^-∆∆CT^ method using GAPDH as a housekeeping gene.

### 2.11. Egg Infectious Dose 50 (EID_50_)

The H9N2 virus (Y280 strain), provided by the Korean National Institute of Environmental Research, was propagated in 10-day-old embryonated, SPF chicken eggs using a standard protocol. The EID_50_ titer was measured and then used for challenge in chickens. Following the challenge, the virus in the lungs of immunized chickens was measured using the EID_50_ titer in the embryonated chicken eggs. Briefly, at 3 days post-challenge, lungs were harvested and homogenized in PBS containing 10 mM HEPES. The samples were 10-fold serially diluted, and 100 μL was inoculated into five eggs per sample. After inoculation, the eggs were sealed with wax and incubated at 35 °C for 2 days. Then the allantoic fluid was harvested, and a hemagglutination test [39] was performed to detect the virus. Using the Reed and Muench method, the viral load was measured by EID_50_ per gram of lung lysate.

### 2.12. Hematoxylin and Eosin (H&E) Staining

Histopathological examination of the spleen, liver, and cecum was performed to assess the potential damage caused by wild-type SG. Three birds from each group were sacrificed on the seventh day post-challenge. The respective organs were harvested and preserved in 10% formalin. The tissues were cut into 4 μm sections and processed for H&E staining following a standard protocol, as described elsewhere [3].

Furthermore, lung tissues from H9N2-challenged birds were harvested on day 7 post-challenge. The same protocol was applied as described earlier for H&E staining to provide detailed insights into the structural and pathological changes.

### 2.13. Statistical Analysis

All data analysis was performed using GraphPad Prism 5.00 software (San Diego, CA, USA). Statistical analyses employed one-way analysis of variance (ANOVA) followed by Tukey’s multiple comparison test for evaluating differences among groups. For datasets involving two independent variables, two-way ANOVA was applied to assess the interaction. The *p*-values < 0.05 were considered significant. Moreover, the results were presented as means ± SD.

## 3. Results

### 3.1. Confirmation of In Vivo Expression of H9N2 Antigens

**Figure 1:**
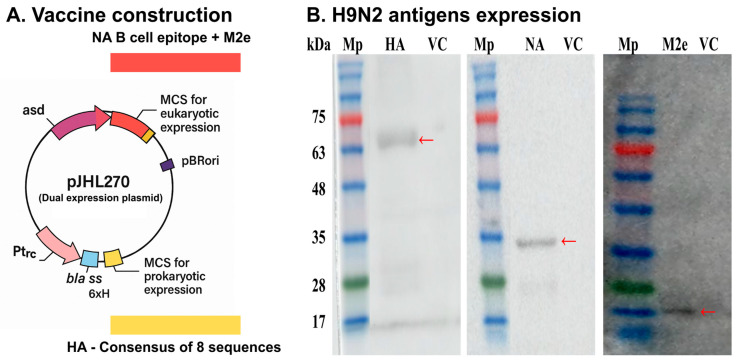
(**A**) A dual expression plasmid was developed to express H9N2 antigens in both Salmonella and eukaryotic cells. For Salmonella expression, HA gene sequences from the H9N2 influenza A virus strains of South Korea were utilized to prepare the HA consensus sequence. For eukaryotic expression, the B cell epitope of NA and the complete M2e sequence linked with furin protease cleavage were cloned for eukaryotic expression. (**B**) In vivo expression of recombinant H9N2 proteins assessed by Western Blot analysis. The expected immunoreactive bands at 63, 32, and 15 kDa indicated efficient expression of HA, NA, and M2e, respectively. No such bands were detected in the splenic tissues of chickens inoculated with JOL3122 (Vector control; VC). Mp: protein marker. Red arrows showing expected bands of antigens.

The designated HA and NA-M2e sequences were cloned into the prokaryotic and eukaryotic regions of pJHL270, respectively (Figure 1A). The expression of HA, NA, and M2e was evaluated in vitro and in vivo in the splenic tissues of the immunized chickens, as confirmed by qRT-PCR and Western blotting, respectively. The expected immunoreactive band at ~63 kDa indicated an efficient expression of HA. The NA-M2e construct was designed as a fusion protein in which the B cell epitope of NA was linked to the M2e via a furin protease cleavage site. Upon in vivo expression, cleavage at this site resulted in separate fragments, corresponding to NA (~32 kDa) and M2e (~15 kDa), as observed in splenic tissues of immunized chickens. No such bands were detected in the protein samples collected from the vector control JOL3122 (Figure 1B). Efficient antigen expression in splenic tissues of immunized chickens confirms that the oral inoculation of therapeutic SG successfully reaches systemic circulation and delivers protective antigens in the host for subsequent immune elicitation.

### 3.2. SG- and H9N2-Specific Antibody Responses

**Figure 2:**
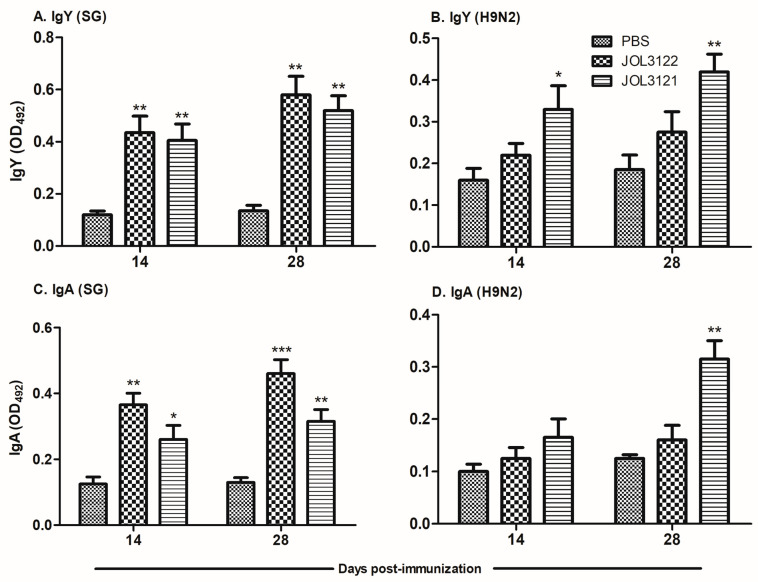
Humoral and mucosal immune responses. Chickens were vaccinated orally with PBS, JOL3122, or JOL3121. Blood and cloacal swab samples were collected on days 14 and 28 post-immunization. (**A**) SG-specific IgY and (**B**) H9N2-specific IgY were measured using serum samples via indirect ELISA at an absorbance of 492 nm. Similarly, (**C**) SG-specific secretory IgA and (**D**) H9N2-specific IgA were assessed using cloacal swab samples. The data were analyzed using two-way ANOVA, with significant differences presented as * *p* < 0.05, ** *p* < 0.01, and *** *p* < 0.001, compared to the PBS control.

The development of IgY and IgA antibody responses against SG and H9N2 was measured in serum and cloacal swab samples collected on days 14 and 28 post-immunization. JOL3122 and JOL3121 strains elicited significantly higher systemic IgY responses against SG (Figure 2A) compared to the PBS control group at both 14 and 28 dpi (*p* < 0.01). Specifically, JOL3122 induced a marginally higher IgY response than JOL3121, although the difference between the two groups was not statistically significant. The development of H9N2-specific antibody responses was initiated by day 14 post-immunization; however, a significant increase could be observed by day 28 post-immunization. Such H9N2-specific antibody responses were not visible in the JOL3122 vector control (Figure 2B). In terms of mucosal immunity, both JOL3122 and JOL3121 significantly enhanced SG-specific IgA levels in cloacal swab samples as early as day 14 post-immunization and increased towards day 28 post-immunization. Regarding H9N2-specific IgA response (Figure 2D), a significant increase was observed only in the JOL3121 group at 28 dpi (*p* < 0.01), indicating effective mucosal immune stimulation. Overall, as anticipated, the JOL3122 effectively induced relatively higher SG-specific humoral and mucosal immune responses, while JOL3121 elicited significantly stronger immune responses against both H9N2 and SG.

### 3.3. Cell-Mediated Immune Responses

**Figure 3:**
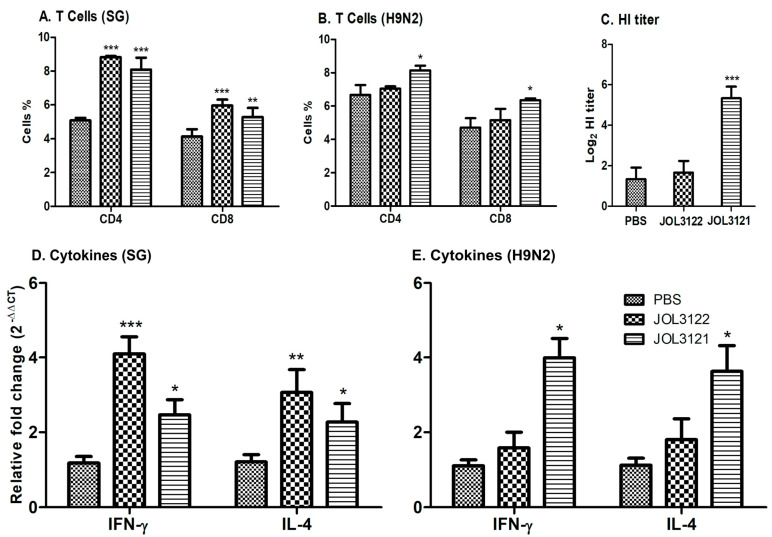
Cellular immune response post-immunization. Flow cytometry was performed to measure the CD4+ and CD8+ T cells within PBMCs isolated from the immunized birds at day 28 post-immunization. (**A**) Bar diagram showing the percentages of CD4+ and CD8+ T cells in the PBMCs of immunized birds after stimulation with SG crude protein. (**B**) Bar diagram showing the CD4+ and CD8+ T cell percentages following stimulation of cells with formalin-inactivated H9N2. (**C**) Hemagglutination inhibition (HI) titer value against 0.75% chicken RBCs. (**D**) Changes in the cytokine expression profiles of IFN-γ and IL-4 in orally immunized chickens were determined by qRT-PCR after restimulating the cells with SG crude protein. (**E**) Cytokine expression profiles of IFN-γ and IL-4 after restimulation with H9N2 inactivated virus. The data were analyzed using one-way ANOVA with Tukey’s multiple comparisons or two-way ANOVA where applicable; with significant differences presented as * *p* < 0.05, ** *p* < 0.01, and *** *p* < 0.001, compared to the PBS control.

Inducing a cellular immune response is considered an important factor for an effective vaccine candidate. Flow cytometry was performed to measure the T lymphocyte subsets, especially CD4+ and CD8+ T cells, within PBMCs isolated from the immunized birds at day 28 post-immunization. Both JOL3121- and JOL3122-immunized groups significantly increased the CD3CD4+ and CD3CD8+ T cells as compared to the PBS control group against SG (Figure 3A). Similarly, regarding H9N2-stimulated T cells, a significant increase (*p* < 0.05) in CD3CD4+ and CD3CD8+ was observed only in the JOL3121 vaccine group (Figure 3B). These results demonstrate that JOL3122 predominantly stimulated T-cell-mediated immunity against SG, while JOL3121 induced a significant cellular response against both H9N2 and SG.

### 3.4. HI Titration

An HI assay was performed using serum samples from all groups collected four weeks post-immunization. The sera obtained from the chickens immunized with JOL3121 had a significantly higher HI titer (*p* < 0.001) compared to the PBS control group (Figure 3C). This finding indicates that the antibodies generated after vaccination probably attach to the receptor-binding sites of AIVs, preventing viral attachment to the host cells.

### 3.5. Cytokine Response

Two weeks after the booster immunization, the cellular immune response was further assessed through Th1 (IFN-γ) and Th2 (IL-4) cytokine gene expression analysis using qRT-PCR. After restimulating the PBMCs with SG crude protein, chickens immunized with JOL3122 and JOL3121 revealed a significant increase in both IFN-γ and IL-4 compared to the PBS control (Figure 3D). In contrast, only the JOL3121-immunized group showed significantly higher levels of IFN-γ and IL-4 expression compared to PBS after restimulation with the H9N2 inactivated virus (Figure 3E). These findings illustrate the potential of our vaccine construct to elicit a balanced Th1/Th2-based antibacterial and antiviral immunological response.

### 3.6. Protection Against Wild-Type SG

**Figure 4:**
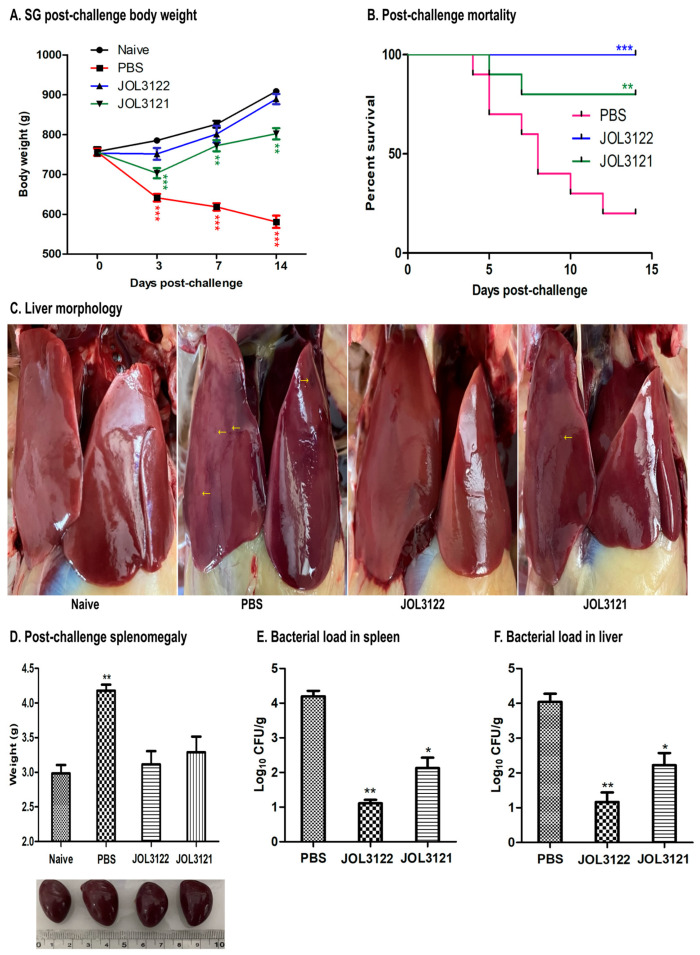
Evaluation of protection upon wild-type SG challenge. (**A**) Body weight changes. Changes in chicken body weight (*n* = 5 per group) were recorded post-challenge to assess the degree of protection against the wild-type challenge. (**B**) Post-challenge mortality. The survival of immunized birds challenged with the wild-type SG was compared with that of the non-immunized group. A Kaplan–Meier survival curve was developed using mortality records till 14 days post-challenge and immunized birds were compared with the PBS group. (**C**) Liver morphology. Morphological changes in the liver (*n* = 5 per group) were examined for hepatic lesions post-challenge. Th yellow colored arrows denote hepatic lesions. (**D**) Splenomegaly and spleen morphology. Post-challenge spleen weights (*n* = 5 per group) were measured and compared with those of naïve birds, and the spleen was examined for splenomegaly and other morphological changes post-challenge. (**E**) Microbial load of the challenge strain in the spleen. (**F**) The bacterial load of the challenge strain in the liver of the birds (*n* = 5 per group). The data were analyzed using one-way ANOVA with Tukey’s multiple comparisons or two-way ANOVA where applicable; with significant differences presented as * *p* < 0.05, ** *p* < 0.01, and *** *p* < 0.001, compared to the control group.

Two weeks after the booster oral immunization, the vaccinated chickens were challenged with wild-type SG (JOL422) to evaluate the protective efficacy achieved post-immunization. The chickens were regularly monitored throughout the experiment, and body weights for all groups were recorded. Following the SG challenge, the PBS group showed significant weight loss compared to the naïve control group. At the same time, those immunized with JOL3122 or JOL3121 maintained comparatively stable and higher body weights during the 14-day post-challenge period (Figure 4A). Chickens in the PBS group exhibited severe clinical signs of fowl typhoid, including greenish diarrhea, lethargy, and increased body temperature. Furthermore, the PBS group experienced 80% mortality, whereas all chickens in the JOL3122 group and 80% in the JOL3121 group survived (Figure 4B). These results indicate that both the JOL3122 and JOL3121 groups were better protected against the lethal SG challenge.

**Figure 5:**
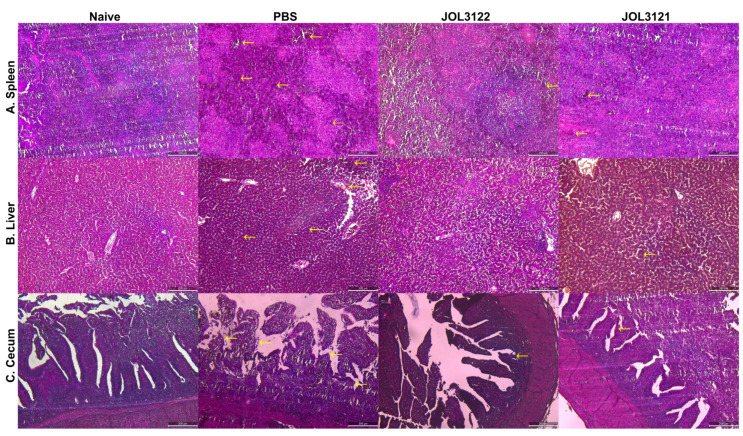
Histopathological changes and microscopic lesions in chickens orally infected with the wild-type SG strain. Histopathological analysis of the internal organs was performed using H&E staining. (**A**) Spleen: Disruption of normal cellular alignment and tissue architecture was evident in spleen sections. In the PBS group, degeneration and necrosis of the white pulp were observed, as indicated by arrows. (**B**) Liver: Liver sections displayed disrupted architecture and inflammatory lesions, along with degeneration and necrosis in the PBS group. Arrows indicate areas of inflammation and congestion. (**C**) Cecum: The PBS group exhibited tissue disruption in the cecum, including shortened and thickened villi, in contrast to the naïve and vaccinated groups. The arrows denote disturbed, thickened and shortened villi. Tissues from uninfected (naïve) chickens served as healthy controls. Data were visualized and analyzed using light microscopy. Scale bar: 200 µm.

A comparative reduction in splenomegaly and lesions of splenic and liver tissue was also observed in the immunized groups compared to the PBS group. Furthermore, the histopathological assessment of the spleen, liver, and cecum indicated a comparative reduction in inflammatory signs in JOL3122 and JOL3121 compared to the PBS group (Figure 5).

### 3.7. Protection Against H9N2 Challenge

**Figure 6:**
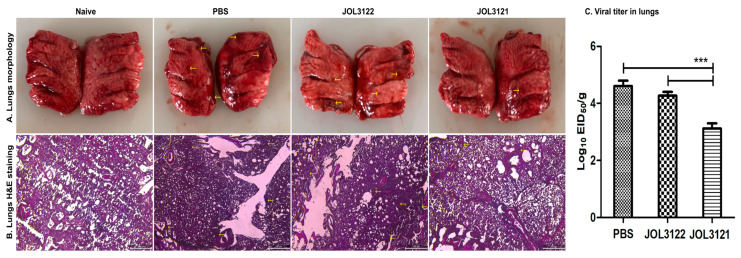
Evaluation of protection against H9N2 challenge. (**A**) Gross pathology of lung samples collected on day 7 post-challenge with H9N2 Y280 influenza A virus. In the challenged PBS and vector control groups, edema, congestion, and hemorrhages were observed. The arrows indicate congestion and hemorrhages. (**B**) Photomicrographs of H&E-stained lung sections from chickens on day 7 post-H9N2 challenge. Samples from the PBS and vector control groups showed inflammatory cell infiltrates, edema, and thickened alveolar walls. The arrows indicate the inflammatory cells and thickening of alveolar walls. Scale bar: 200 µm. (**C**) Viral load in lungs after challenge. The viral load in chicken (*n* = 5 per group) lung samples was measured by EID_50_/g using the Reed and Muench method. The data were analyzed using one-way ANOVA with Tukey’s multiple comparisons, with significant differences presented as *** *p* < 0.001, compared to the PBS and vector control.

Two weeks after the booster inoculation, all experimental groups were challenged with the H9N2 Y280 strain. On days 3 and 7 post-challenge, four birds from each group were euthanized to evaluate pathological signs and protective efficacy. The JOL3121 group showed minimal to no clinical signs, while the PBS and JOL3122 (VC) groups exhibited pronounced respiratory signs, including coughing, sneezing, gasping, rales, and rattles. Upon post-mortem examination, immunized birds in the JOL3121 group showed no significant lung congestion or hemorrhages compared to the PBS and JOL3122 groups (Figure 6A). The histopathological examination of lung samples collected from PBS and JOL3122 (VC) groups showed pronounced inflammatory responses, likely due to the viral challenge (Figure 6B). Moreover, birds immunized with JOL3121 showed significantly reduced viral titer in their lungs compared to the PBS and JOL3122 groups (Figure 6C). These findings indicate that, while the immunized birds (JOL3121) were better protected than those in the PBS and JOL3122 (VC) groups, they still exhibited some observable pathological changes.

## 4. Discussion

The present study was aimed at investigating the immunoprotective efficacy of SG-based H9N2 oral vaccine against FT and H9N2 infection in chickens. The continuous genetic variation and reassortment of the circulating viral strains are a major obstacle in developing an effective influenza vaccine [40,41]. Moreover, the frequent spillover of low-pathogenic avian influenza A (H9N2) virus lineages into pigs and humans raises concern about its pandemic potential. In addition to its zoonotic transmission, H9N2 also acts as a genetic contributor to the emergence of highly pathogenic zoonotic influenza viruses, such as H5Nx and H7N9 [42,43,44,45]. Furthermore, currently available vaccines provide little to no cross-protection against heterologous strains [38]. These global concerns highlight the urgent need for a universal vaccine with highly antigenic and conserved proteins.

Hemagglutinin (HA) is a crucial glycoprotein, composed of HA1 and HA2 subunits linked by a disulfide bond. The HA1 subunit contains the receptor-binding domain for viral attachment, while HA2 facilitates membrane fusion during the viral entry into the host cells [30,31,46]. Thus, HA is a primary target for inducing potent neutralizing antibodies against avian influenza virus (AIV) [32]. We selected a consensus sequence of HA from South Korean H9N2 Y280 circulating strains that could induce neutralizing antibodies and prevent the viral attachment by blocking interaction with the sialic acid receptor on the host cells [42,47]. The more conserved NA and M2e offer a broader range of cross-protection across heterologous strains [48]. Therefore, combining HA, NA, and M2e antigens in a vaccine construct could enhance immunoprotective responses against H9N2. Moreover, this strategy could minimize the need for frequent vaccine updates due to continuous genetic variations.

Subunit and mRNA vaccines stimulate immune responses through extracellular and intracellular antigen delivery, respectively [49,50]. We developed a combined strategy by using a dual expression plasmid, pJHL270, with Ptrc and CMV promoters to stimulate immune responses via extracellular and intracellular antigen expression [51]. The HA consensus sequence was cloned in prokaryotic regions of pJHL270 to enable exogenous presentation from *Salmonella*, activating T helper cells. On the other hand, NA-M2e was delivered via endogenous expression from the host cells, leading to the activation of CD8+ cytotoxic cells (Figure 1A). To deliver and express the plasmid containing H9N2 antigens (pJHL270: pHA + eNA-M2e), we developed an attenuated SG mutant strain with ∆*lon* ∆*pagL* ∆*asd* deletions. The Lon protease is a key global regulator of virulent genes, and its deletion reduces *Salmonella* virulence while maintaining immunogenicity [47,48,49]. The *pagL* gene encodes 3-O-deacylase, which is involved in TLR-4-MD2-dependent LPS recognition and *Salmonella* pathogenesis. It was reported that *pagL*-mediated deacylation reduced lipid-A-mediated NF-κB secretion through the TLR-4 signaling pathway and endotoxicity [51,52]. It was reported that these modifications enhanced the vaccine safety and immunogenicity while lowering the endotoxicity level [3,52,53,54]. Moreover, we have reported earlier that the aforementioned SG mutant did not create any adverse effects on the immunized birds and reduced the proinflammatory cytokine secretions, confirming its safety and reduced endotoxicity [3]. Moreover, these alterations offer a protective background against FT, adding to its practical advantages in field applications.

The expression of each H9N2 antigen by the *Salmonella* delivery system was confirmed in vitro by qRT-PCR and in vivo in the splenic tissues of chickens by Western blot (Figure 1B). It shows that the oral inoculation of JOL3121 successfully reaches systemic circulation and delivers protective H9N2 antigens in the chickens for subsequent immune elicitation.

Adaptive immunity can have an antiviral effect, especially IgY, which reduces viral pneumonia, while IgA prevents viral infection in the upper respiratory tract and clears epithelial viral infection [55,56]. In this study, we demonstrated that oral immunization with the JOL3121 vaccine strain induced a significant increase in H9N2-specific systemic and mucosal immune responses as compared to PBS control (Figure 2B,D). These results correlate with the protection efficacy acquired via oral immunization with JOL3121, producing a high hemagglutination inhibition titer and exhibiting a satisfactory level of protection against H9N2 Y280 challenge in the chickens.

T-cell-mediated immune responses also play a critical role in cross-protection against the influenza virus [55]. CD4+ T cells recognize the viral peptide and stimulate B cells to produce neutralizing antibodies. Moreover, they promote CD8+ T cells to trigger the apoptosis of infected cells via cytotoxic T lymphocytes. We have demonstrated that the chickens immunized with JOL3121 had significantly higher cell-mediated immunity as compared to the PBS control group (Figure 3B). This kind of immunomodulation was further supported by elevated secretion of Th1 (IFN-γ) and Th2 (IL-4) cytokines in the JOL3121-immunized group (Figure 3E). The IFN-γ secretion stimulates cell-mediated immunity by activating cytotoxic CD8+ cells [57,58], while IL-4 supports B cells to produce antibodies [59]. As the vaccine construct had a dual promoter vector system, it effectively promoted Th1- and Th2-based immune responses. Our results align with the previous findings that this type of vaccination strategy induces Th1/Th2-based mixed immunity consistent with humoral and cellular immune responses. After challenge with H9N2 Y280 strain, chickens immunized with the JOL3121 vaccine strain exhibited a significant decrease in viral load compared to the PBS and vector control (JOL3122) groups. The lung tissue morphology underscored the protective efficacy of the JOL3121 vaccine (Figure 6A). Moreover, negligible inflammatory cell infiltrates and thickening of alveolar walls were observed in the immunized chickens (Figure 6B). The immunized chickens demonstrated a robust decrease in lung lysate viral titer as compared to PBS and vector controls (JOL3122), further corroborating the protective efficacy of our JOL3121 vaccine strain against H9N2 (Figure 6C).

We further validated that the vaccine strains could induce an immunoprotective response against FT. Assessment of both humoral and mucosal immunity demonstrated that oral immunization significantly enhanced the levels of SG-specific IgY and IgA. As mucosal surfaces act as an immunological barrier for enteric pathogens like *Salmonella*, IgA antibodies prevent the adherence and invasion, enabling the clearance of *Salmonella* from the intestinal epithelium of host cells [60,61]. Cell-mediated immune responses, particularly T cells (CD4+ and CD8+) and immunomodulatory cytokine production, are essential for the clearance of intracellular pathogens such as *Salmonella* [62]. We confirmed that our vaccine strains elicited a significant humoral and cell-mediated immunity against SG, particularly JOL3122 (vector control), which effectively induced a relatively higher SG-specific immune response than JOL3121. After challenge with wild-type SG (JOL422), chickens immunized with JOL3122 and JOL3121 showed a significant reduction in spleen and liver bacterial load (Figure 4E,F). Moreover, JOL3122 (empty vector) and JOL3121 demonstrated 100% and 80% protection, respectively, against FT. This survival difference against SG may be due to the antigenic burden of H9N2 in the JOL3121 strain, which could suppress the protection against lethal wild-type SG challenge. We observed that the vaccine strain is slightly slower in growth compared to the vector control. This could affect the survival rate of the *Salmonella* strain within the host, leading to a lower rate of protection against the SG challenge. This scenario is reflected in our results, indicating slightly low antibody and protective responses against the SG virulent challenge. Previous studies also showed that recombinant *Salmonella* vectors expressing foreign antigens could display reduced colonization ability, lower growth rate, and be cleared rapidly from host tissues [63,64].

## 5. Conclusions

We employed a dual expression plasmid vector system, pJHL270, containing both prokaryotic and eukaryotic promoters to express exogenous and endogenous HA and NA-M2e antigens derived from H9N2 avian influenza virus (AIV). Utilizing an engineered *Salmonella* Gallinarum strain as a delivery vehicle, this strategy effectively elicited robust humoral, mucosal, and cell-mediated immune responses, ensuring a satisfactory protection level with minimal adverse effects. These results demonstrate that our SG-based H9N2 vaccine candidate has promising potential as an immunoprotective vaccine against H9N2 and FT in chickens.

## Figures and Tables

**Table 1:** List of bacterial strains, plasmids, and primers.

Bacteria/Primers	Description	References
*S*. Gallinarum		
JOL422 JOL2997	Wild type Δ*lon* Δ*pagL*	Lab stock Lab stock [3]
JOL3062	Δ*lon* Δ*pagL* Δ*asd*	Lab stock
JOL3121	pJHL270 (pro HA + eu NA-M2e) in JOL3062	This study
JOL3122	pJHL270 empty vector in JOL3062	This study
Plasmid		
pJHL270 pET28a (+)	*asd*+, CMV eukaryotic promoter, Ptrc prokaryotic promoter, BlaSS secretory signal sequence, pBR322 ori IPTG-inducible expression vector; kanamycin resistant	Lab stock Novagen, USA
Primers		
IFN-γ	Forward: CAAAGCCGCACATCAAACA Reverse: TTTCACCTTCTTCACGCCAT	Lab stock
IL-4	Forward: GGAGAGCATCCGGATAGTGA Reverse: TGACGCATGTTGAGGAAGAG	Lab stock
GAPDH HA NA M2e	Forward: AGAACATCATCCCAGCGTCC Reverse: CGGCAGGTCAGGTCAACA Forward: AGAATGGAAACCATTCTGGTGGTG Reverse: TTACGCAATCACCAGGCTGCT Forward: CTTGGGCAGGGAACCA Reverse: AGCCCACCCTTTCACT Forward: ATGAGTCTTCTAACCGAG Reverse: TTACTCCAGCTCTA	Lab stock This study This study This study

**Table 2:** Experiment schedule for immunization and challenge.

Group	Route	Dosage (CFU/200 µL)	Booster Dosage (CFU/200 µL)	SG Challenge (CFU/200 µL, Oral)	H9N2 Challenge (EID_50_/100 µL, Intranasal)
PBS	Oral	200 µL	200 µL	1 × 10^6^	1 × 10^6^
JOL3122	Oral	1 × 10^8^	1 × 10^8^	1 × 10^6^	1 × 10^6^
JOL3121	Oral	1 × 10^8^	1 × 10^8^	1 × 10^6^	1 × 10^6^
Naïve	-	-	-	-	-

## Data Availability

The data reported in the manuscript can be made available upon reasonable request from the corresponding authors.

## References

[1] Peacock T.H.P., James J., Sealy J.E., Iqbal M.A. (2019). Global Perspective on H9N2 Avian Influenza Virus. Viruses.

[2] Hu Z., Ai H., Wang Z., Huang S., Sun H., Xuan X., Chen M., Wang J., Yan W., Sun J. (2025). Impact of inactivated vaccine on transmission and evolution of H9N2 avian influenza virus in chickens. NPJ Vaccines.

[3] Aganja R.P., Bakhsh M., Senevirathne A., Kwon J., Lee J.H. (2025). Lipopolysaccharide structural modification in *Salmonella* Gallinarum targeting lipid a deacylation and O-antigen reduces virulence and endotoxicity with competitive protection against a wild-type challenge in chickens. Dev. Comp. Immunol..

[4] Bin Aslam H., Hasler B., Iqbal M., Yaqub T., Alarcon P. (2024). Financial impact of low pathogenic avian influenza virus subtype H9N2 on commercial broiler chicken and egg layer production systems in Pakistan. Prev. Vet. Med..

[5] Moatasim Y., Kandeil A., Mostafa A., Elghaffar S.K.A., El Shesheny R., Elwahy A.H.M., Ali M.A. (2017). Single gene reassortment of highly pathogenic avian influenza A H5N1 in the low pathogenic H9N2 backbone and its impact on pathogenicity and infectivity of novel reassortant viruses. Arch. Virol..

[6] Sun Y., Liu J. (2015). H9N2 influenza virus in China: A cause of concern. Protein Cell.

[7] Ali S., Robie E.R., Saeed U., Jaffar G., Bailey E.S., Marushchak L.V., Kreditor B.E., Pulscher L.A., Rubrum A.M., Webby R.J. (2024). H9N2 influenza A viruses found to be enzootic in Punjab Pakistan’s bird markets with evidence of human H9N2 nasal colonization. Int. J. Infect. Dis..

[8] Zhang J., Huang L., Liao M., Qi W. (2023). H9N2 avian influenza viruses: Challenges and the way forward. Lancet Microbe.

[9] Peiris M., Yuen K.Y., Leung C.W., Chan K.H., Ip P.L., Lai R.W., Orr W.K., Shortridge K.F. (1999). Human infection with influenza H9N2. Lancet.

[10] Guo J., Wang Y., Zhao C., Gao X., Zhang Y., Li J., Wang M., Zhang H., Liu W., Wang C. (2021). Molecular characterization, receptor binding property, and replication in chickens and mice of H9N2 avian influenza viruses isolated from chickens, peafowls, and wild birds in eastern China. Emerg. Microbes Infect..

[11] Kilany W.H., Bazid A.H., Ali A., El-Deeb A.H., El-Abideen M.A., Sayed M.E., El-Kady M.F. (2016). Comparative Effectiveness of Two Oil Adjuvant-Inactivated Avian Influenza H9N2 Vaccines. Avian Dis..

[12] Swayne D.E., Kapczynski D. (2008). Strategies and challenges for eliciting immunity against avian influenza virus in birds. Immunol. Rev..

[13] Zhao Y., Li S., Zhou Y., Song W., Tang Y., Pang Q., Miao Z. (2015). Phylogenetic Analysis of Hemagglutinin Genes of H9N2 Avian Influenza Viruses Isolated from Chickens in Shandong, China, between 1998 and 2013. Biomed. Res. Int..

[14] Zhao J., Yang H., Xu H., Ma Z., Zhang G. (2017). Efficacy of an inactivated bivalent vaccine against the prevalent strains of Newcastle disease and H9N2 avian influenza. Virol. J..

[15] Alves Batista D.F., de Freitas Neto O.C., Maria de Almeida A., Maboni G., de Carvalho T.F., de Carvalho T.P., Barrow P.A., Berchieri A.J. (2018). Evaluation of pathogenicity of *Salmonella* Gallinarum strains harbouring deletions in genes whose orthologues are conserved pseudogenes in *S*. Pullorum. PLoS ONE.

[16] Kwon Y.K., Kim A., Kang M.S., Her M., Jung B.Y., Lee K.M., Jeong W., An B.K., Kwon J.H. (2010). Prevalence and characterization of *Salmonella* Gallinarum in the chicken in Korea during 2000 to 2008. Poult. Sci..

[17] Kim N.H., Ha E.J., Ko D.S., Lee C.Y., Kim J.H., Kwon H.J. (2019). Molecular evolution of *Salmonella* enterica subsp. enterica serovar Gallinarum biovar Gallinarum in the field. Vet. Microbiol..

[18] Shivaprasad H.L. (2000). Fowl typhoid and pullorum disease. Rev. Sci. Tech..

[19] Aganja R.P., Kwon J., Senevirathne A., Lee J.H. (2025). Deletion of pagL and arnT genes involved in LPS structure and charge modulation in the *Salmonella* genome confer reduced endotoxicity and retained efficient protection against wild-type *Salmonella* Gallinarum challenge in chicken. Vet. Res..

[20] Ntakiyisumba E., Tanveer M., Won G. (2024). Integrating meta-analysis with a quantitative microbial risk assessment model to investigate Campylobacter contamination of broiler carcasses. Food Res. Int..

[21] Desin T.S., Koster W., Potter A.A. (2013). *Salmonella* vaccines in poultry: Past, present and future. Expert. Rev. Vaccines.

[22] Wigley P., Hulme S., Powers C., Beal R., Smith A., Barrow P. (2005). Oral infection with the *Salmonella* enterica serovar Gallinarum 9R attenuated live vaccine as a model to characterise immunity to fowl typhoid in the chicken. BMC Vet. Res..

[23] Lee Y.J., Mo I.P., Kang M.S. (2007). Protective efficacy of live *Salmonella* gallinarum 9R vaccine in commercial layer flocks. Avian Pathol..

[24] Van Immerseel F., Studholme D.J., Eeckhaut V., Heyndrickx M., Dewulf J., Dewaele I., Van Hoorebeke S., Haesebrouck F., Van Meirhaeghe H., Ducatelle R. (2013). *Salmonella* Gallinarum field isolates from laying hens are related to the vaccine strain SG9R. Vaccine.

[25] Mukhtar M., Ghafoor A., McClelland M., Akhtar F., Rasheed M.A. (2024). Construction, molecular characterization, and safety assessment of purB mutant of *Salmonella* Gallinarum. Front. Microbiol..

[26] Galen J.E., Wahid R., Buskirk A.D. (2021). Strategies for Enhancement of Live-Attenuated *Salmonella*-Based Carrier Vaccine Immunogenicity. Vaccines (Basel).

[27] Roberts M., Bacon A., Li J., Chatfield S. (1999). Prior immunity to homologous and heterologous *Salmonella* serotypes suppresses local and systemic anti-fragment C antibody responses and protection from tetanus toxin in mice immunized with *Salmonella* strains expressing fragment C. Infect. Immun..

[28] Hajam I.A., Kirthika P., Hewawaduge C., Jawalagatti V., Park S., Senevirathne A., Lee J.H. (2020). Oral immunization with an attenuated *Salmonella* Gallinarum encoding the H9N2 haemagglutinin and M2 ectodomain induces protective immune responses against H9N2 infection in chickens. Avian Pathol..

[29] Ntakiyisumba E., Tanveer M., Won G. (2025). A comprehensive analysis of the current strategy for developing live attenuated vaccines against African swine fever: A systematic review and meta-analysis. Vaccine.

[30] Sun Y., Cong Y., Yu H., Ding Z., Cong Y. (2021). Assessing the effects of a two-amino acid flexibility in the Hemagglutinin 220-loop receptor-binding domain on the fitness of Influenza A(H9N2) viruses. Emerg. Microbes Infect..

[31] Russell C.J. (2021). Hemagglutinin Stability and Its Impact on Influenza A Virus Infectivity, Pathogenicity, and Transmissibility in Avians, Mice, Swine, Seals, Ferrets, and Humans. Viruses.

[32] Zhang Y., Xu C., Zhang H., Liu G.D., Xue C., Cao Y. (2019). Targeting Hemagglutinin: Approaches for Broad Protection against the Influenza A Virus. Viruses.

[33] Jagadesh A., Salam A.A., Mudgal P.P., Arunkumar G. (2016). Influenza virus neuraminidase (NA): A target for antivirals and vaccines. Arch. Virol..

[34] Mezhenskaya D., Isakova-Sivak I., Rudenko L. (2019). M2e-based universal influenza vaccines: A historical overview and new approaches to development. J. Biomed. Sci..

[35] Deng L., Cho K.J., Fiers W., Saelens X. (2015). M2e-Based Universal Influenza A Vaccines. Vaccines (Basel).

[36] Guo Y., He L., Song N., Li P., Sun S., Zhao G., Tai W., Jiang S., Du L., Zhou Y. (2017). Highly conserved M2e and hemagglutinin epitope-based recombinant proteins induce protection against influenza virus infection. Microbes Infect..

[37] Bradford M.M. (1976). A rapid and sensitive method for the quantitation of microgram quantities of protein utilizing the principle of protein-dye binding. Anal. Biochem..

[38] World Organisation for Animal Health (OIE) Manual of Diagnostic Tests and Vaccines for Terrestrial Animals; 2008. https://www.woah.org/fileadmin/Home/eng/Health_standards/tahm/A_summry.htm.

[39] Killian M.L. (2014). Hemagglutination assay for influenza virus. Methods Mol. Biol..

[40] Nuwarda R.F., Alharbi A.A., Kayser V. (2021). An Overview of Influenza Viruses and Vaccines. Vaccines (Basel).

[41] Yamayoshi S., Kawaoka Y. (2019). Current and future influenza vaccines. Nat. Med..

[42] Wang J., Wu M., Hong W., Fan X., Chen R., Zheng Z., Zeng Y., Huang R., Zhang Y., Lam T.T. (2016). Infectivity and Transmissibility of Avian H9N2 Influenza Viruses in Pigs. J. Virol..

[43] Nagy A., Mettenleiter T.C., Abdelwhab E.M. (2017). A brief summary of the epidemiology and genetic relatedness of avian influenza H9N2 virus in birds and mammals in the Middle East and North Africa. Epidemiol. Infect..

[44] Bhat S., James J., Sadeyen J.R., Mahmood S., Everest H.J., Chang P., Walsh S.K., Byrne A.M.P., Mollett B., Lean F. (2022). Coinfection of Chickens with H9N2 and H7N9 Avian Influenza Viruses Leads to Emergence of Reassortant H9N9 Virus with Increased Fitness for Poultry and a Zoonotic Potential. J. Virol..

[45] Iqbal M., Yaqub T., Reddy K., McCauley J.W. (2009). Novel genotypes of H9N2 influenza A viruses isolated from poultry in Pakistan containing NS genes similar to highly pathogenic H7N3 and H5N1 viruses. PLoS ONE.

[46] Myers M.L., Gallagher J.R., Kim A.J., Payne W.H., Maldonado-Puga S., Assimakopoulos H., Bock K.W., Torian U., Moore I.N., Harris A.K. (2023). Commercial influenza vaccines vary in HA-complex structure and in induction of cross-reactive HA antibodies. Nat. Commun..

[47] Dascalu S., Sealy J.E., Sadeyen J.R., Flammer P.G., Fiddaman S., Preston S.G., Dixon R.J., Bonsall M.B., Smith A.L., Iqbal M. (2024). Immunisation of chickens with inactivated and/or infectious H9N2 avian influenza virus leads to differential immune B-cell repertoire development. Front. Immunol..

[48] Wei C.J., Crank M.C., Shiver J., Graham B.S., Mascola J.R., Nabel G.J. (2020). Next-generation influenza vaccines: Opportunities and challenges. Nat. Rev. Drug Discov..

[49] Chen S., Pounraj S., Sivakumaran N., Kakkanat A., Sam G., Kabir M.T., Rehm B.H.A. (2023). Precision-engineering of subunit vaccine particles for prevention of infectious diseases. Front. Immunol..

[50] Liu T., Liang Y., Huang L. (2021). Development and Delivery Systems of mRNA Vaccines. Front. Bioeng. Biotechnol..

[51] Aganja R.P., Kim I.S., Tae H.J., Lee J.H. (2025). Expression and delivery of HA1-M2e antigen using an innovative attenuated *Salmonella*-mediated delivery system confers promising protection against H9N2 avian influenza challenge. Poult. Sci..

[52] Park B.S., Song D.H., Kim H.M., Choi B.S., Lee H., Lee J.O. (2009). The structural basis of lipopolysaccharide recognition by the TLR4-MD-2 complex. Nature.

[53] Senevirathne A., Hewawaduge C., Sivasankar C., Lee J.H. (2022). Prospective lipid-A altered live attenuated *Salmonella* Gallinarum confers protectivity, DIVA capability, safety and low endotoxicity against fowl typhoid. Vet. Microbiol..

[54] Kong Q., Six D.A., Roland K.L., Liu Q., Gu L., Reynolds C.M., Wang X., Raetz C.R., Curtiss R. (2011). 3rd. Salmonella synthesizing 1-monophosphorylated lipopolysaccharide exhibits low endotoxic activity while retaining its immunogenicity. J. Immunol..

[55] Ito R., Ozaki Y.A., Yoshikawa T., Hasegawa H., Sato Y., Suzuki Y., Inoue R., Morishima T., Kondo N., Sata T. (2003). Roles of anti-hemagglutinin IgA and IgG antibodies in different sites of the respiratory tract of vaccinated mice in preventing lethal influenza pneumonia. Vaccine.

[56] Suzuki T., Kawaguchi A., Ainai A., Tamura S., Ito R., Multihartina P., Setiawaty V., Pangesti K.N., Odagiri T., Tashiro M. (2015). Relationship of the quaternary structure of human secretory IgA to neutralization of influenza virus. Proc. Natl. Acad. Sci. USA.

[57] Hemann E.A., Kang S.M., Legge K.L. (2013). Protective CD8 T cell-mediated immunity against influenza A virus infection following influenza virus-like particle vaccination. J. Immunol..

[58] Whitmire J.K., Tan J.T., Whitton J.L. (2005). Interferon-gamma acts directly on CD8+ T cells to increase their abundance during virus infection. J. Exp. Med..

[59] Macaulay A.E., DeKruyff R.H., Goodnow C.C., Umetsu D.T. (1997). Antigen-specific B cells preferentially induce CD4(+) T cells to produce IL-4. J. Immunol..

[60] Duerkop B.A., Vaishnava S., Hooper L.V. (2009). Immune responses to the microbiota at the intestinal mucosal surface. Immunity.

[61] Endt K., Stecher B., Chaffron S., Slack E., Tchitchek N., Benecke A., Van Maele L., Sirard J.C., Mueller A.J., Heikenwalder M. (2010). The microbiota mediates pathogen clearance from the gut lumen after non-typhoidal *Salmonella* diarrhea. PLoS Pathog..

[62] Ismail N., Olano J.P., Feng H.M., Walker D.H. (2002). Current status of immune mechanisms of killing of intracellular microorganims. Fems Microbiol. Lett..

[63] Wolfenden R.E., Layton S.L., Wolfenden A.D., Khatiwara A., Gaona-Ramirez G., Pumford N.R., Cole K., Kwon Y.M., Tellez G., Bergman L.R. (2010). Development and evaluation of candidate recombinant *Salmonella*-vectored *Salmonella* vaccines. Poult. Sci..

[64] Wang S., Li Y., Scarpellini G., Kong W., Shi H., Baek C.H., Gunn B., Wanda S.Y., Roland K.L., Zhang X. (2010). *Salmonella* vaccine vectors displaying delayed antigen synthesis in vivo to enhance immunogenicity. Infect. Immun..

